# Metagenomics and metaproteomics alterations are associated with kidney disease in opisthorchiasis hamsters fed a high-fat and high-fructose diet

**DOI:** 10.1371/journal.pone.0301907

**Published:** 2024-05-30

**Authors:** Keerapach Tunbenjasiri, Thatsanapong Pongking, Chutima Sitthirach, Suppakrit Kongsintaweesuk, Sitiruk Roytrakul, Sawanya Charoenlappanit, Sirinapha Klungsaeng, Sirirat Anutrakulchai, Chalongchai Chalermwat, Chawalit Pairojkul, Somchai Pinlaor, Porntip Pinlaor

**Affiliations:** 1 Biomedical Science Program, Graduate School, Khon Kaen University, Khon Kaen, Thailand; 2 Chronic Kidney Disease Prevention in Northeastern Thailand, Khon Kaen University, Khon Kaen, Thailand; 3 Department of Parasitology, Faculty of Medicine, Khon Kaen University, Khon Kaen, Thailand; 4 Centre for Research and Development of Medical Diagnostic Laboratories, Faculty of Associated Medical Sciences, Khon Kaen University, Khon Kaen, Thailand; 5 Functional Proteomics Technology Laboratory, National Center for Genetic Engineering and Biotechnology, National Science and Technology Development Agency, Pathum Thani, Thailand; 6 Department of Medicine, Faculty of Medicine, Khon Kaen University, Khon Kaen, Thailand; 7 Department of Biochemistry, Faculty of Medicine, Khon Kaen University, Khon Kaen, Thailand; 8 Department of Pathology, Faculty of Medicine, Khon Kaen University, Khon Kaen, Thailand; 9 Department of Microbiology, Faculty of Associated Medical Sciences, Khon Kaen University, Khon Kaen, Thailand; Niigata University of Pharmacy and Medical and Life Sciences, JAPAN

## Abstract

**Background:**

*Opisthorchis viverrini* (*O*. *viverrini*, Ov) infection and consumption of high-fat and high-fructose (HFF) diet exacerbate liver and kidney disease. Here, we investigated the effects of a combination of *O*. *viverrini* infection and HFF diet on kidney pathology via changes in the gut microbiome and host proteome in hamsters.

**Methodology/Principal findings:**

Twenty animals were divided into four groups; 1) fed a normal diet not infected with *O*. *viverrini* (normal group), 2) fed an HFF diet and not infected with *O*. *viverrini* (HFF), 3) fed a normal diet and infected with *O*. *viverrini* (Ov), and 4) fed an HFF diet and infected with *O*. *viverrini* (HFFOv). DNA was extracted from fecal samples and the V3–V4 region of the bacterial 16S rRNA gene sequenced on an Illumina MiSeq sequencing platform. In addition, LC/MS-MS analysis was done. Histopathological studies and biochemical assays were also conducted. The results indicated that the HFFOv group exhibited the most severe kidney injury, manifested as elevated KIM-1 expression and accumulation of fibrosis in kidney tissue. The microbiome of the HFFOv group was more diverse than in the HFF group: there were increased numbers of *Ruminococcaceae*, *Lachnospiraceae*, *Desulfovibrionaceae* and *Akkermansiaceae*, but fewer *Eggerthellaceae*. In total, 243 host proteins were identified across all groups. Analysis using STITCH predicted that host proteome changes may lead to leaking of the gut, allowing molecules such as soluble CD14 and p-cresol to pass through to promote kidney disease. In addition, differential expression of TGF-beta-activated kinase 1 and MAP3K7-binding protein 2 (Tab2, involving renal inflammation and injury) are predicted to be associated with kidney disease.

**Conclusions/Significance:**

The combination of HFF diet and *O*. *viverrini* infection may promote kidney injury through alterations in the gut microbiome and host proteome. This knowledge may suggest an effective strategy to prevent kidney disease beyond the early stages.

## Introduction

Chronic kidney disease (CKD) is defined as abnormalities in both the structure and/or function of the kidney. The increasing incidence and prevalence of CKD are major health concerns, impacting approximately 10% of the global population [1]. In Thailand, a prevalence of 17.5% was reported in 2009, with the highest at 22.2% in the northeastern region. CKD is believed to be a multifactorial disease with risk factors that include increasing age, gender, local genetic background, diabetes mellitus and hypertension [2].

Infection with the small liver fluke, *Opisthorchis viverrini*, (*O*. *viverrini*, Ov) [3, 4] or *Strongyloides stercoralis*, consumption of monosodium glutamate or high-fat and high-fructose (HFF) diet change the composition of the gut microbiome (gut dysbiosis), thus contributing to the development of various diseases including CKD [5, 6]. *Opisthorchis viverrini* infects at least six million people, with the highest prevalence noted in Northeast Thailand. Long-term intake of HFF diet [7, 8] and chronic *O*. *viverrini* infection [9] can cause renal pathology in animal models. We anticipate that the combination of both risk factors (chronic *O*. *viverrini* infection and bad diet) will enhance the severity of kidney pathology [10]. Thus, understanding the underlying mechanism by which these risk factors combine to promote kidney disease would be of benefit for limiting CKD progression, particularly in endemic areas of *O*. *viverrini* infection.

A previous study revealed that a combination of *O*. *viverrini* infection and a HFF diet is associated with liver and kidney pathology [11]. However, whether this combination actually causes CKD is still unclear. In particular, the role of these factors in altering the gut microbiota and proteome remains unclear. Gut dysbiosis has a key role in many inflammatory-related diseases. It can increase the permeability of the intestinal mucosal barrier leading to entry of inflammatory factors and endotoxins into the bloodstream [12–14]. Many previous studies have reported gut dysbiosis associated with CKD both of humans and animals [6, 15]. Liquid chromatography tandem mass spectrometry (LC-MS/MS) technologies have the capacity to provide deeper information for both identification and quantification of proteins in fecal samples [16, 17]. Proteomics-based approaches can improve understanding of the pathophysiological mechanisms of CKD and identification of new CKD-related biomarkers [18]. A combination of metagenomics and metaproteomics technologies would aid exploration of the pathophysiology of renal dysfunction and provide insights into treatment and prevention of CKD [19, 20].

In this study, we hypothesized that a combination of *O*. *viverrini* infection and consumption of a HFF diet promotes kidney injury via changes in the gut microbiome. In addition, we used metaproteomics to try to identify biomarkers associated with kidney injury. The results of this study may point towards an effective strategy to prevent the progression of CKD.

## Materials and methods

### Ethics statement

The samples in this study were used in a previous study (IACUC–KKU–81/62) [21]. All of the experimental protocols were reviewed and approved by the Animal Ethics Committee of Khon Kaen University (IACUC–KKU (C)-88/65) based on Ethics of Animal Experimentation of National Research Council of Thailand. Hamsters were obtained from the Animal Unit, Faculty of Medicine, Khon Kaen University.

### Experimental design and sample collections

Twenty male Syrian golden hamsters (*Mesocricetus auratus*: 4–6 weeks old and 80–100 g weight) were used. Hamsters were divided into 4 groups (n = 5 each) and all hamsters were reared and maintained under the same conditions. To avoid bacterial contamination, the stainless-steel cages were washed once a week with Sunlight detergent (Unilever, Thailand), decontaminated using the antimicrobial reagent Dettol (Dettol, Thailand) and sawdust was changed twice per week. The groups and abbreviations used were as follows: 1) fed a normal diet and not infected with *O*. *viverrini* (normal group), 2) fed an HFF diet and not infected with *O*. *viverrini* (HFF group), 3) fed a normal diet and infected with *O*. *viverrini* (Ov group), and 4) fed an HFF diet and infected with *O*. *viverrini* (HFFOv group).

The HFF diet consisted of standard hamster diet (40%, Smart Heart, Thailand), coconut oil (10%, Roi Thai, Thailand), corn oil (10%, Golden drop, Thailand), cholesterol (1.25%, Sigma-Aldrich, St, Louis, Mo, USA), sodium deoxycholate (0.25%, Sigma-Aldrich), and sucrose (38.5%, Mitrphol, Thailand). The ingredient of the high-fat diet was determined by Central Laboratory (Thailand), Co. Ltd. using Association of Official Analytical Chemists, AOAC. To the drinking water of the HFFOv group was added d-glucose (final concentration 1.89%) and 2 d-fructose (final concentration 2.31%).

Metacercariae of *O*. *viverrini* were obtained from naturally infected cyprinid fish from Khammouane Province, Lao PDR. All fish were digested in 0.25% of pepsin-HCl solution. After that the sediments were examined under a stereomicroscope to identify and collect active metacercariae. Finally, the metacercariae were fed to all infected hamster groups by gastric intubation. Each hamster was infected with 50 metacercariae [21].

Animals were sacrificed at the end of the three-month experiment. Each animal was anesthetized using isoflurane 0.05 mL/ chamber 1 L before being sacrificed by drawing blood from the heart aseptically.

**Fig 1:**
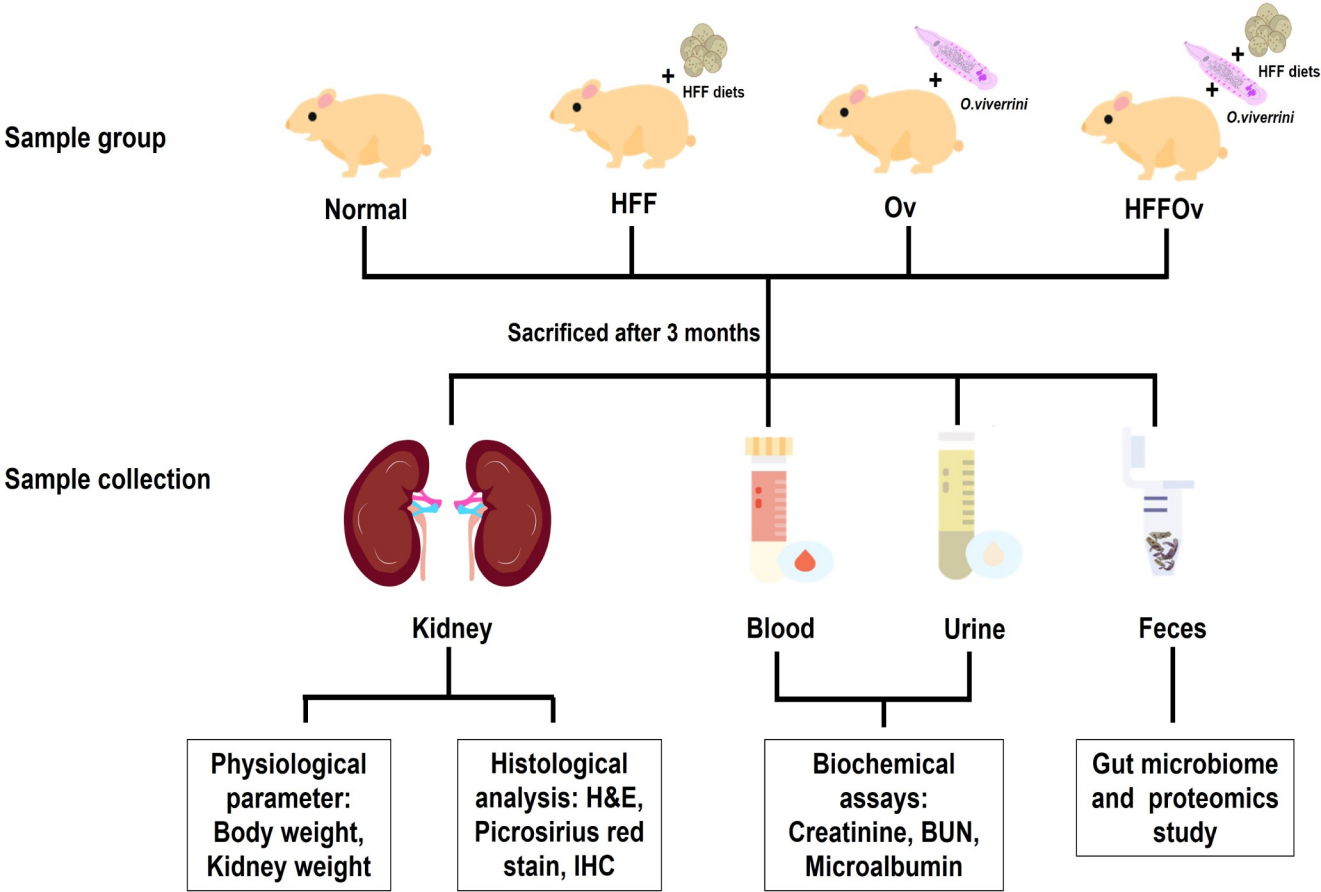
Experimental design. Abbreviations are as follow: 1) fed a normal diet not infected with *O*. *viverrini* (normal group), 2) fed an HFF diet and not infected with *O*. *viverrini* (HFF group), 3) fed a normal diet and infected with *O*. *viverrini* (Ov group), and 4) fed an HFF diet and infected with *O*. *viverrini* (HFFOv group).

Serum and urine samples from each animal were stored at -20°C until used to determine biochemical kidney functions. A portion of each kidney sample was preserved in 10% buffered formalin for histopathological and immunohistochemical analysis. Fecal samples were snap frozen in liquid nitrogen for 16S-rRNA next-generation sequencing (16S-rRNA NGS) for gut microbiome analysis and for proteomics analysis using liquid chromatography with tandem mass spectrometry (LC-MS/MS), as shown in (**Fig 1)**.

### Measurement of serum/urine biochemistry parameters

Blood and urine biochemical parameters relating to kidney function, including blood urea nitrogen (BUN), serum creatinine (SCr) and urine albumin to creatinine ratio (ACR), were measured using the automated analyzers (Cobas 8000 Modular Analysis Series, Roche Diagnostics, Mannheim, Germany) at the clinical laboratory section, Srinagarind Hospital, Faculty of Medicine, Khon Kaen University, Khon Kaen, Thailand.

### Histopathological investigation of kidney changes

To investigate the histopathology of kidneys from the various groups (tubular damage, inflammatory cell infiltration, and tubular fibrosis), staining with Mayer’s hematoxylin and eosin (H&E) and picrosirius red were used. After being fixed in 10% buffered formalin overnight, kidney tissues were cut and embedded in paraffin wax using an automated machine. The paraffin-embedded kidney tissues were cut into 5 μm sections using a microtome. To stain with H&E, the sections were deparaffinized in xylene then rehydrated in descending concentrations of ethanol (100%, 95% and 70%, three times for each concentration) and finally in water for 5 minutes. After rehydration, each slide was stained with Mayer’s hematoxylin for 8 minutes and eosin for 5 minutes and was washed twice with distilled water. The slides were then dehydrated in ascending concentrations of ethanol (70%, 95% and 100%, three times for each concentration) and finally cleared in xylene. Stained slides were mounted using mounting media and air-dried overnight at room temperature. Histopathology was observed under a light microscope.

We used picrosirius red staining to show the extent of fibrosis. Each tissue section was incubated at 100°C for 3 minutes and deparaffinized in xylene for 5 minutes (three changes) to remove paraffin wax. Following rehydration as for H&E, each section was rinsed in tap water for 5 minutes, stained with Mayer’s hematoxylin for 8 minutes and washed in running tap water for 1 minute. The slide was then stained with picrosirius red (Solution A for 2 minutes, Solution B for 90 minutes and Solution C for 2 minutes). Slides were then dehydrated and mounted as for H&E. Collagen stained with picrosirius red is red on a pale-yellow background. The severity of kidney pathology was graded based on the EGTI histology scoring system for alterations in tubular, endothelial, glomerular, and tubulointerstitial regions [22]. Grades of kidney pathology (**S1 Fig**) were assigned (double-blind) by two authors and the consistency of the results was confirmed by a senior pathologist.

### Immunohistochemical study

Immunohistochemistry was used to show localization of proteins in kidney sections. Paraffin-embedded kidney tissue sections were deparaffinized in xylene and were rehydrated in descending concentrations of ethanol (100%, 95% and 70%, three times for each concentration) and finally in water for 5 minutes. After rehydration, antigen was unmasked by autoclaving with sodium citrate buffer (10 mM sodium citrate, 0.05% Tween 20, pH 6.0). Thereafter, slides were immersed in 3% H_2_O_2_ for 30 minutes to quench endogenous peroxidase. Then, non-specific binding was blocked by incubating the slides with 5% fetal bovine serum (FBS) for 30 minutes at room temperature. The sections were incubated with mouse monoclonal anti-kidney injury molecule-1 (KIM-1) antibody (NBP1-76701, Novus biologicals, CO, USA; 1:150 dilution) diluted in 1% FBS at 4°C overnight. The next day, the slides were washed with phosphate buffered saline solution (PBS) followed by incubation with horseradish peroxidase (HRP)-conjugated secondary antibodies for goat anti-rabbit (111-035-003; Jackson Immunoresearch, PA, USA; 1:200 dilution) diluted in 1% FBS at room temperature for 2 hours. Finally, the immunoreactivity was developed by adding 3,3-diaminobenzidine [23] and slides were counterstained with Mayer’s hematoxylin for 2 minutes. 1% FBS was used as a negative control instead of primary antibodies. Kidney sections of adenine-induced kidney disease were used as positive controls [24]. Ten representative, randomly chosen areas were monitored from the Aperio ImageScope software (Leica biosystems, IL, USA) using 40X magnification. The percentage area of the cell-injury marker (KIM-1) was determined using ImageJ software (National Institutes of Health, Bethesda, MD, USA) [25].

### 16S-rRNA next-generation sequencing analysis

#### Fecal DNA extraction

Fecal samples from twenty hamsters were subjected to microbial DNA extraction using the QIAamp PowerFecal Pro DNA Kit (Qiagen, Hilden, Germany) according to the manufacturer’s instructions. DNA concentration of each sample was measured by Nanodrop2000 spectrophotometer (Thermo Scientific, MA, USA). After extraction and checking, all DNA samples were stored at -20°C until analysis.

#### 16S rRNA gene sequencing and analysis

The V3-V4 region of the bacterial 16S-rRNA gene was PCR-amplified individually from each hamster using a BioRad C1000TM Thermal Cycler (Biorad, CA, USA). PCR primers used and cycling conditions were as previously reported [3]. Gel electrophoresis (1.5% agarose) was used to confirm the size of the product, which should be approximately 450–500 bp.

The 16S ribosomal RNA (rRNA) gene was sequenced using an Illumina MiSeq platform with paired-end reads (2×300bp) (Illumina Inc., CA, USA). Before each sequencing run, standard Illumina protocols were followed for sterilization and washing of the machine. Raw sequencing data were processed using LotuS2 (version 1.65) [26, 27], with default settings applied throughout to filter input sequences based on parameters such as average quality, accumulated error, quality within a user-defined window, and removal of 5′ low-quality bases. Additionally, sequences underwent filtering for minimum/maximum length, ambiguous nucleotides, maximum barcode and primer errors, polynucleotide runs, and trimming of adapter sequences if present. Filtered sequences were then clustered into Operational Taxonomic Units (OTUs) using UPARSE version v11.0 followed by alignment against a reference 16S ribosomal DNA (rDNA) with SILVA SSU database release 138 (https://www.arb-silva.de/download/arb-fles/) as reference and the default similarity threshold set at 0.90. Taxonomic consistency was assessed at each taxonomic level, with a taxonomic assignment accepted by default if over 90% of references matched the same taxon. References lacking taxonomic information at a given level were discarded.

Microbiome diversity analysis was conducted using R software version 4.3.0 [28]. Alpha diversity, indicating species diversity within a sample, was evaluated by Shannon and Simpson indices, using phyloseq package version 1.44.0; ggboxplot was used for alpha diversity plotting and statistical comparison by ggpubr package version 0.6.0. For beta diversity analysis, which examines differences in species composition among samples, The PCoA of Bray-Curtis distance was computed using the vegan package version 2.6.4, along with weighted UniFrac values calculated using the UniFrac method from the phyloseq package version 1.44.0. Statistical comparison between sample groups was performed using permutational multivariate analysis of variance (PERMANOVA). Principal component analysis (PCA) plots were generated using cmdscale from the stats package version 4.3.0. Relative abundance was calculated using the phyloseq package version 1.44.0, and stacked bar-plots were created based on relative abundance values, with prevalence filtering: 10%, using ggplot2 version 3.4.4. Additionally, a heat map was constructed based on the relative abundance value of each microbial taxon which was calculated from row Z-score and was generated using the pheatmap package version 1.0.12. The pipeline of microbiome analysis was shown in **S1 File**.

### Metaproteomics analysis

#### Sample preparation for shotgun proteomics

200 mg of feces from each hamster was homogenized separately with 500 μl of 0.5% sodium dodecyl sulfate (SDS) or lysis buffer and then mixed well by vortexing and centrifuged at 10,000 g for 15 minutes. All supernatants from each sample were transferred to a new tube, mixed well with 1,000 μl of cold acetone, and held overnight at -20°C. The supernatant was removed from the mixture after centrifugation at 10,000 g for 15 minutes. After precipitation, the protein pellet was dried and stored at -80°C until analysis.

The Lowry assay determines protein concentration using bovine serum albumin as the standard protein [29]. Dithiothreitol (10 mM in 10 mM ammonium bicarbonate) was used to break down disulfide bonds, followed by alkylation with 30 mM iodoacetamide in 10 mM ammonium bicarbonate to prevent reforming of disulfide bonds in the proteins. The protein samples were digested using sequencing-grade porcine trypsin for 16 h at 37°C. The tryptic peptides were dried with a speed vacuum concentrator and resuspended in 0.1% formic acid for analysis by nano-liquid chromatography tandem mass spectrometry (nano LC-MS/MS).

#### Liquid chromatography‑tandem mass spectrometry (LC/MS-MS)

Tryptic peptide samples were analyzed using the Thermo Scientific Ultimate3000 Nano/Capillary LC System (Thermo Scientific). It was connected to a hybrid quadrupole Q-Tof impact II (Bruker Daltonics; MA, USA) with a nano-captive spray ion source. Peptide digests were packed with Acclaim PepMap RSLC C18, 2 m, 100, and nanoViper after being enriched on a pre-column 300 m i.d. X 5 mm C18 Pepmap 100, 5 m, 100 A (Thermo Scientific). The C18 column was surrounded by a thermostated column oven set at 60°C. On the analytical column, solvents A (containing 0.1% formic acid in water) and B (containing 0.1% formic acid in 80% acetonitrile) were provided.

Peptides were eluted for 30 minutes at a constant flow rate of 0.30 l/min with a gradient of 5–55% solvent B. CaptiveSpray was used for 1.6 kV electrospray ionization. About 50 l/h of nitrogen was employed as the drying gas. Collision-induced-dissociation (CID) product ion mass spectra were obtained using nitrogen gas as the collision gas. Mass spectra in positive ion mode and MS/MS spectra were obtained at 2 Hz over the range (m/z) 150–2200. Based on the m/z value, the collision energy was changed to 10 eV. The LC-MS analysis was performed three times with each sample.

#### Metaproteomics analysis of samples

Maxquant 2.1.0.0 was used to quantify and identify host proteins in fecal samples. The Uniprot database, accessed July 18, 2022, was used for this purpose. Label-free quantification with default MaxQuant settings was performed using a maximum of two missed cleavages, a mass tolerance of 0.6 daltons for the main search, trypsin as the digestive enzyme, carbamidomethylation of cysteine as a fixed modification, and oxidation of methionine and acetylation of the protein N-terminus as variable modifications. Peptides with at least seven amino acids and at least one unique peptide were considered for protein identification and used for further data analysis. All proteins were found in at least 50% of the samples in each group. The median number of modifications per peptide was set at 5. For each peptide, the median intensity from three injections was determined and used for data analysis of the total proteins expressed by the hamster host.

The peptide peak intensities were transformed in Microsoft Excel, resulting in protein expression levels for quantification of protein number (protein ID) and analysis of differentially expressed host proteins (DEPs). Any protein found in at least 50% of samples in each group was selected for analysis of highly expressed proteins (HEPs) using the Jvenn viewer. All peaks with a significantly higher intensity relative to controls were considered unique and used for the Gene Ontology study by matching the unique protein ID with the Uniprot database. MetaboAnalyst version 5.0 is a comprehensive, freely available web-based metabolomics analysis platform (https://www.metaboanalyst.ca/, accessed September 14, 2023) used for proteomics analysis. The database STITCH, version 5, was used to predict functional interaction networks between identified proteins and biomolecules and to generate highest confidence (0.900) numbers (http://stitch.embl.de/, accessed September 18, 2023).

### Statistical analysis

Statistical analysis was performed using SPSS version 29 (SPSS Inc, IL, USA), GraphPad Prism version 8.4 (GraphPad software, MA, USA), and Excel. All data were expressed as mean ± SD. Analysis of variance (ANOVA) and Tukey’s test were used to test for differences between experimental groups. The staining intensity of proteins in each group was determined using the median value in Excel (Microsoft, WA, USA).

### Data availability

The raw sequencing data have been deposited with the Mendeley Database accession (doi: 10.17632/8hrf8hsx8d.1). The MS/MS raw data and analysis files have been deposited with the ProteomeXchange Consortium (http://proteomecentral.proteomexchange.org) via the jPOST partner repository (https://jpostdb.org) with the data set identifier JPST002317 and PXD045309 (preview URL for reviewers: https://repository.jpostdb.org/preview/2126542412650078fd5e120, Access key: 7273).

## Results

### Effect of HFF diet combined with *O*. *viverrini* infection on biochemical parameters

In this study, there was no significant difference in body weight (BW), kidney weight, and KW /BW ratio among all groups of hamsters. However, the body weight of hamsters in the Ov and HFFOv groups showed an upward trend. Clinical biochemical parameters were analyzed to identify HFFOv-induced changes in renal function and/or pathological features. The Ov and HFFOv groups had significantly lower blood urea nitrogen (BUN) (P < 0.05) and serum creatinine (SCr) levels (P < 0.05) than the normal and HFF groups. The microalbumin to creatinine ratio (ACR) was significantly higher in the Ov and HFFOv groups, as shown in **Table 1**.

**Table 1:** Body weight, kidney weight, kidney weight/body weight ratio and biochemical parameters of hamsters.

Parameter(s)	Normal	HFF	Ov	HFFOv
Body weight (g)	149±6.63	147.6±11.60	172.25±19.27	173.4±16.68
Kidney weight (g)	1.28±0.18	1.10±0.12	1.48±0.26	1.37±0.37
KW/BW (mg/g)	8.56±1.00	7.44±0.95	8.56±0.80	7.91±1.87
BUN (mg/dL)	35.8±7.50	27.08±3.82	22.7±1.32*	19.54±0.97*
SCr (mg/dL)	0.31±0.02	0.31±0.04	0.20±0.03*^,#^	0.24±0.02 *^,#^
ACR (mg/gCre)	2.41±4.17	33.67±28.50	92.35±4.38*	49.69±28.84*

Kidney weight/body weight: KW/BW, blood urea nitrogen: BUN, serum creatinine: SCr, microalbumin to creatinine ratio: ACR. Mean±SD was reported for each parameter. Statistical analyses were performed using one-way ANOVA (P < 0.05) with the Tukey’s HSD post-hoc test and GraphPad Prism version 8.4. *P < 0.05 compared with NC, ^#^P < 0.05 compared with HFF.

### The combination of HFF diet with *O*. *viverrini* infection induced kidney fibrosis

**Fig 2:**
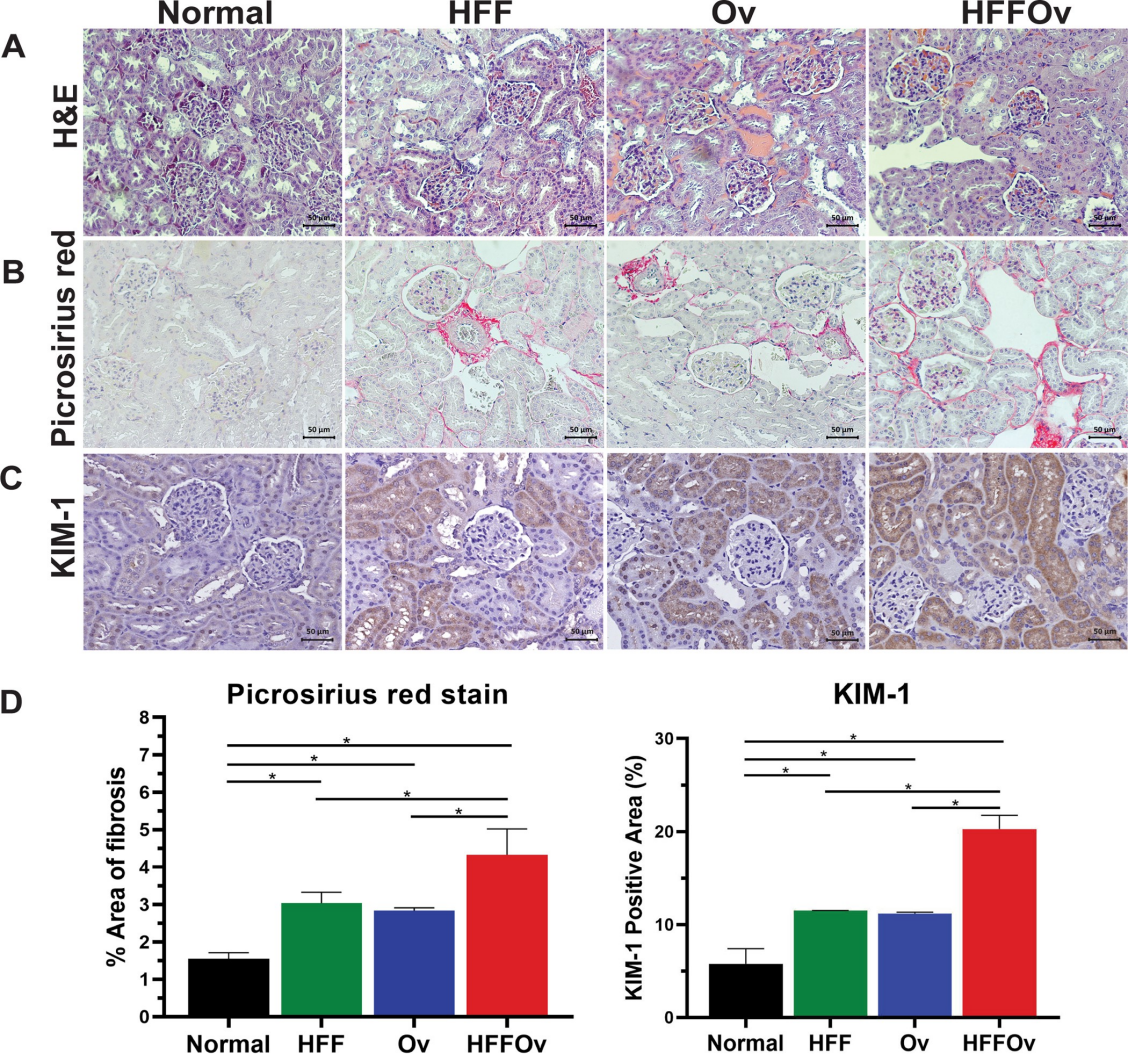
Representative photographs of sections of hamster kidneys. (A) H&E staining (B) picrosirius red staining for fibrosis of kidney tissue, (C) expression of kidney injury molecule-1 (KIM-1) and (D) percentage of picrosirius red staining and KIM-1 positive cells. Results are expressed as mean±SD. Statistical analyses were performed using one-way ANOVA *P < 0.05 (Tukey’s multiple comparisons test) (n = 3 in each group) and GraphPad Prism version 8.4. Scale bar = 50 μm.

Histopathological examination (**Fig 2**) showed representative areas of H&E, KIM-1, and picrosirius-red staining in the kidney tissue. H&E staining of kidney sections (**Fig 2A**) clearly showed that there was mild to moderate tubular dilatation along with the appearance of vacuoles in the glomerulus in the HFF and Ov groups compared with the normal group. The HFFOv group showed yet more severe histopathological changes of the tubular, endothelial, glomerular, and tubulointerstitial regions. Based on the EGTI scoring system, the highest score for kidney pathology was found in the HFFOv group (grade 3.75), followed by the Ov (2.5), HFF (2.0), and normal (0) groups in descending order. The score for the HFFOv group was significantly greater than for the HFF and normal groups (P <0.05), but not for the Ov group (P > 0.05). Moreover, the HFFOv group had a statistically significantly higher area positive for kidney fibrosis as indicated by picrosirius red (**Fig 2B and 2D**). This result was consistent with the staining of the marker KIM-1 (P < 0.05) (**Fig 2C and 2D**), as shown in **Fig 2 and S2 and S3 Figs**.

### The combination of HFF diet and *O*. *viverrini* infection disturbs the gut microbiota

**Fig 3:**
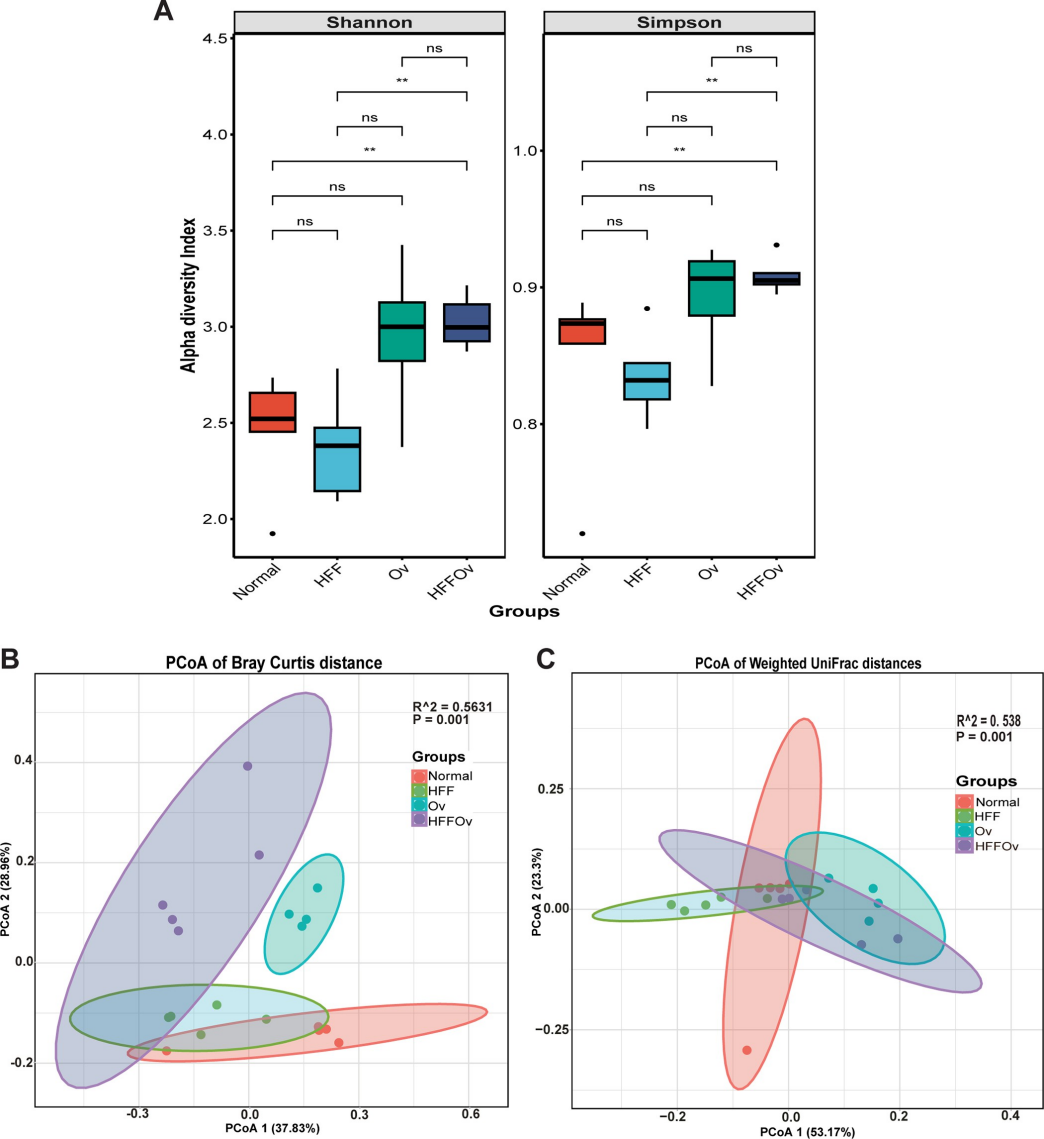
Microbial diversity. (A) Alpha diversity of groups compared at baseline in normal, HFF, Ov and HFFOv groups presented as boxplots for Shannon and Simpson indices. Beta diversity of samples in each group of 16S rRNA sequences demonstrates the distribution of samples as orange dots (Normal), green dots (HFF), blue dots (Ov), and purple dots (HFFOv) in (B) PCoA of Bray-Curtis dissimilarity and (C) PCoA of weighted-Unifrac distances with P-values.

Total reads ranged from 151,189 to 186,207 per sample. An average of 168,384 high-quality sequences were produced from each hamster fecal sample. The gut microbiome was evaluated by Illumina MiSeq sequencing. The Shannon index, which reflects both the species richness and evenness, and the Simpson index, which reflects only species evenness, were used to determine diversity within experimental groups. The alpha diversity of the HFFOv group was significantly higher than that of the normal group (**Fig 3A**). Principal coordinates analysis (PCoA) based on Bray-Curtis distance and weighted UniFrac distance was used to determine beta diversity between experimental groups (**Fig 3B and 3C)**. The results showed a clear separation between the groups, indicating that the microbial communities are different in all groups (P <0.05).

**Fig 4:**
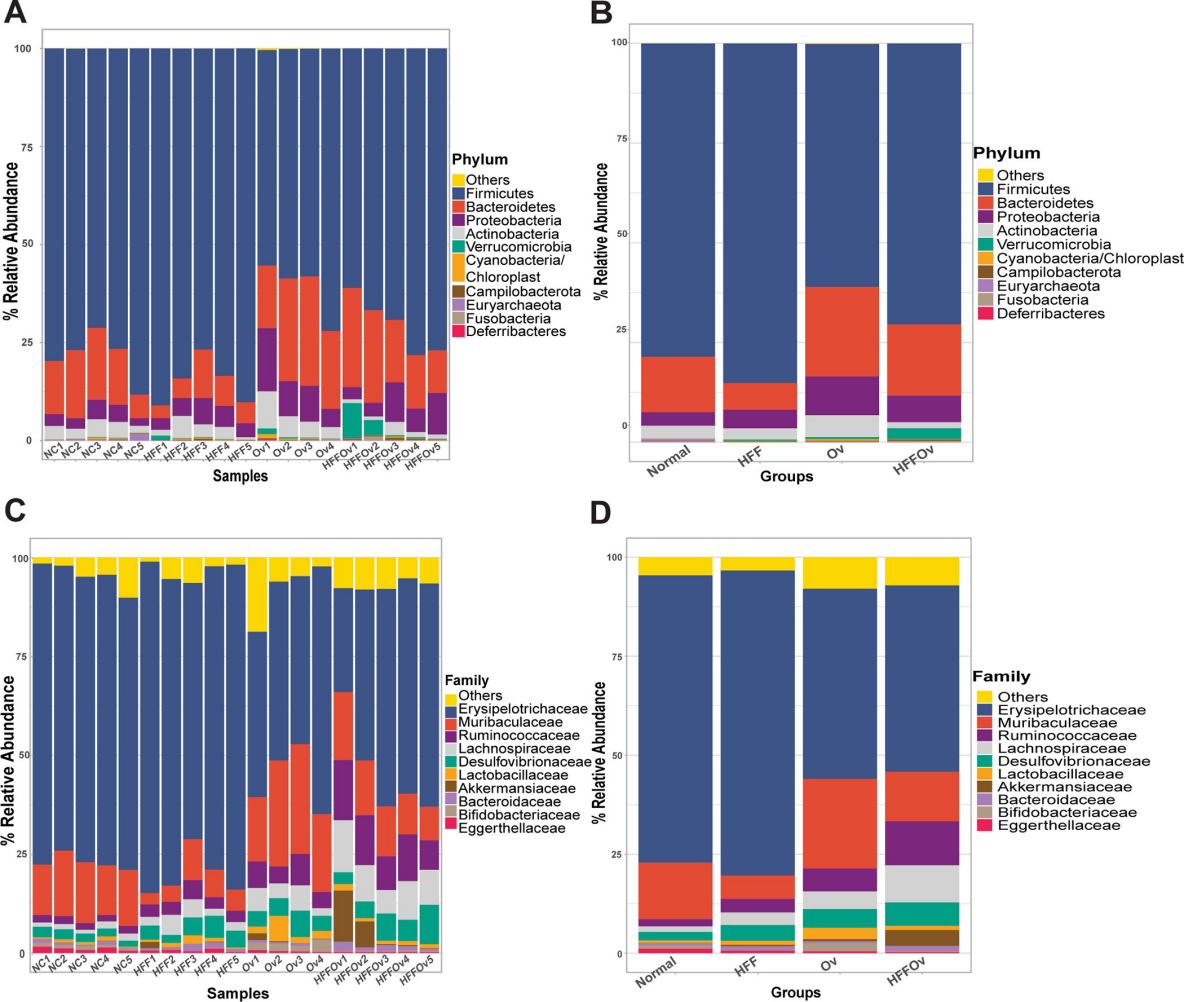
Relative abundance of fecal microbial composition in hamsters of bacterial taxa at the phylum and family levels. Phylum level (A) in each sample and (B) in each group. Family level (C) in each sample and (D) in each group. Sample name is on the x-axis and relative abundance on the y-axis.

*Firmicutes* was the most abundant phylum in all groups (**Fig 4A and 4B**). Phylum *Verrucomicrobia* was very abundant in the HFFOv group, whereas phylum *Actinobacteria* decreased in this group. At the family level (**Fig 4C and 4D**), *Erypsipelotrichaceae* was the most abundant in all groups and showed a trend to decrease in the HFFOv group compared to the normal group. Families *Ruminococcaceae*, *Lachnospiraceae*, *Desulfovibrionaceae* and *Akkermansiaceae* were very abundant in the HFFOv group, while *Eggerthellaceae* decreased.

**Fig 5:**
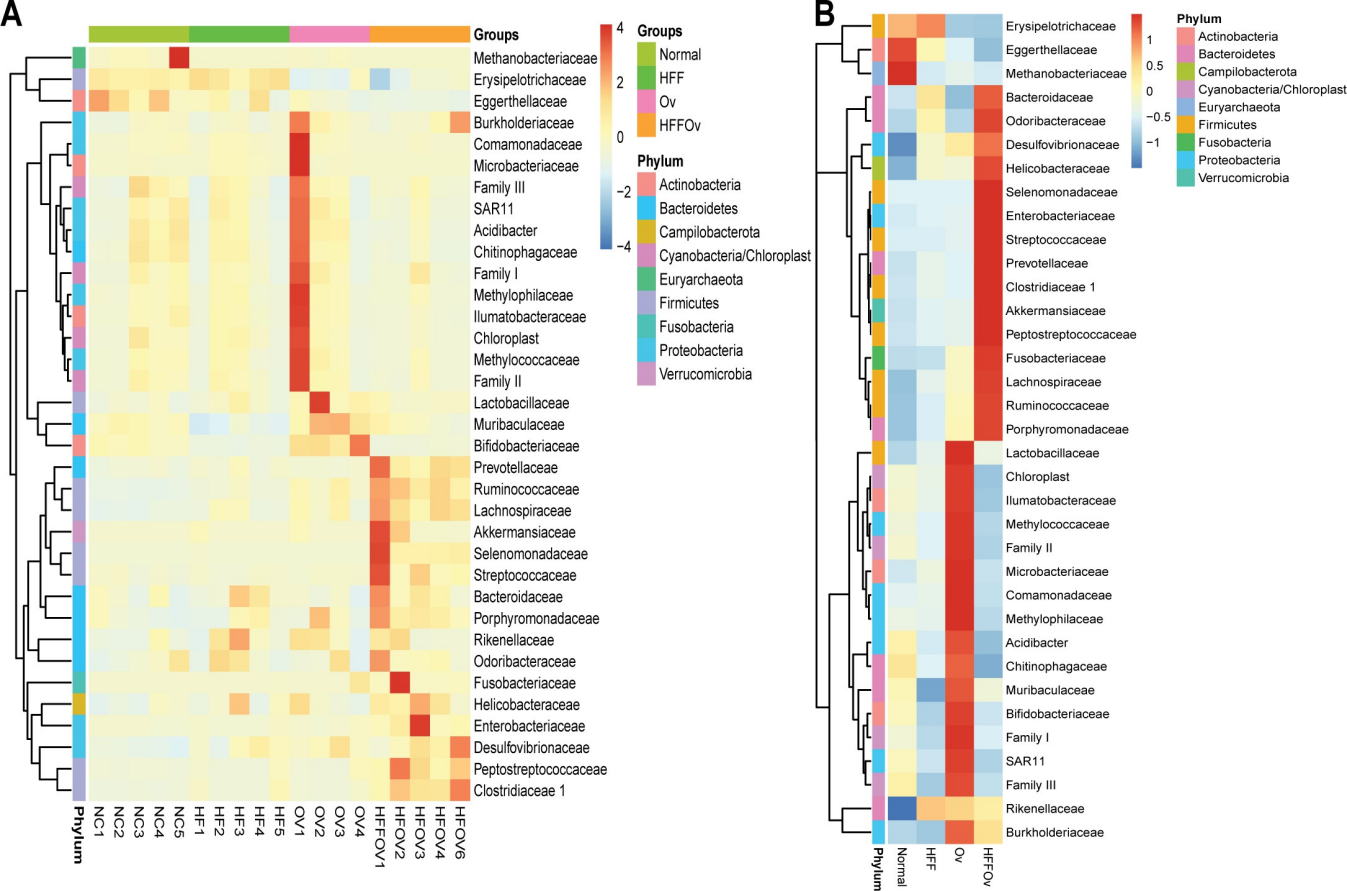
Heat map of the top 35 bacterial families identified in hamster fecal samples. The abundance of bacterial taxa at the family level (A) in each sample and (B) in each group. Both figures were generated using unsupervised hierarchical cluster analysis (blue, low abundance; red, high abundance).

A heatmap of the 35 major families observed in all groups is shown in (**Fig 5**). The results demonstrated that *Eggerthellaceae* and *Methanobacteriaceae* were more abundant in the normal group compared to HFF, Ov and HFFOv. In contrast, the families *Desulfovibrionaceae*, *Helicobacteraceae*, *Selenomonadaceae*, *Enterobacteriaceae*, *Streptococcaceae*, *Prevotellaceae*, *Clostridiaceae* 1, *Akkermansiaceae*, *Peptostreptococcaceae*, *Fusobacteriaceae*, *Lachnospiraceae*, *Ruminococcaceae*, and *Porphyromonadaceae* were very abundant in the HFFOv group and decreased significantly in the Ov, HFF, and normal groups.

### Differentially expressed proteins in the HFFOv group

**Fig 6:**
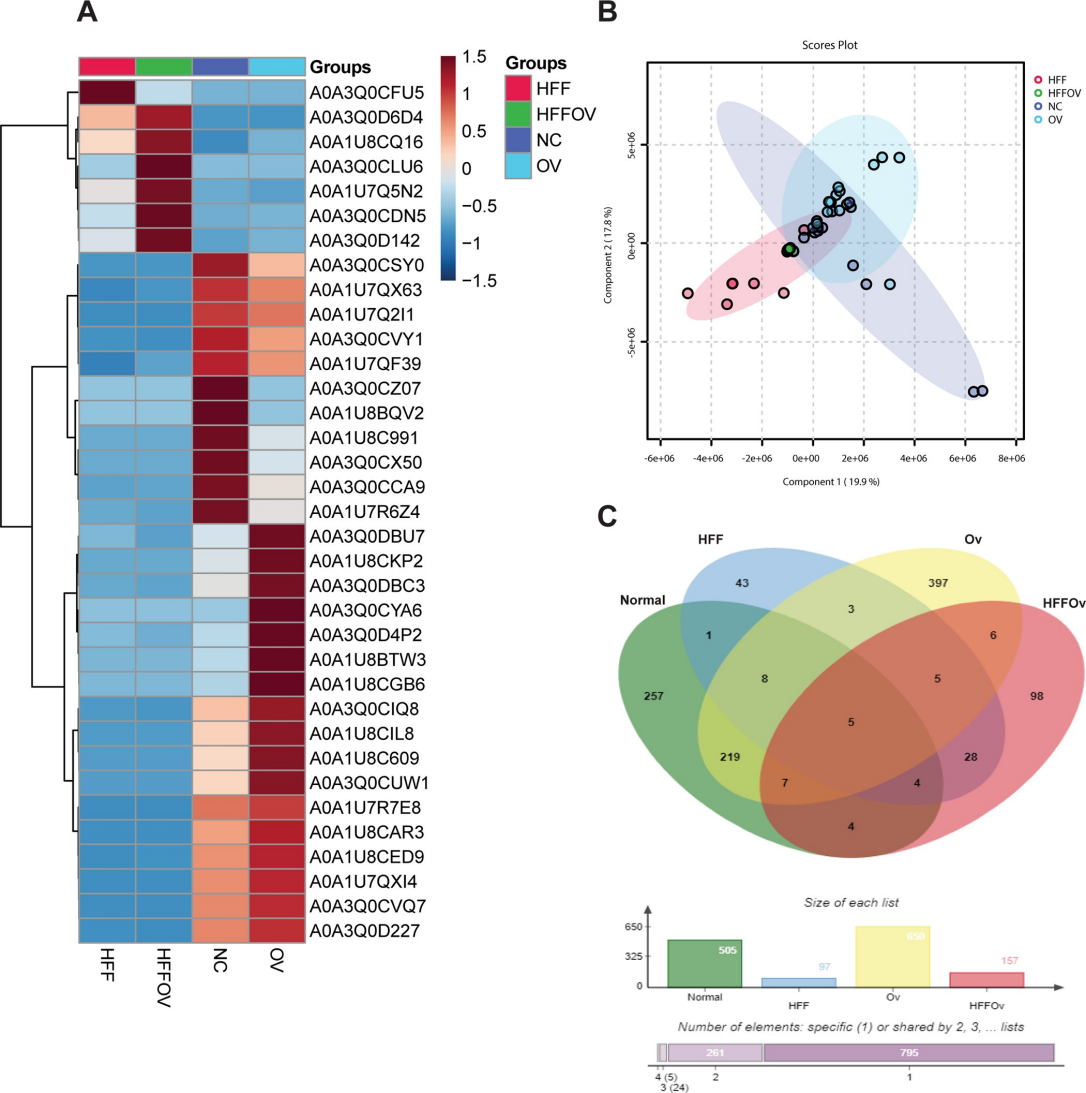
Metaproteomics analysis results. (A) Heatmap of the 35 most abundant host proteins. (B) PLS-DA score plot shows slightly difference between groups. Color dots indicate as follows: red dots (HFF), green dots (HFFOv), dark blue dots (Ov), and light blue dots (Normal). (C) Venn diagram depicting the number of unique proteins in the HFFOv group.

A total of 14,693 host proteins was identified across all groups, of which 243 were significantly differentially expressed proteins (DEPs) based on MetaboAnalyst software. The top 35 most abundant significantly DEPs in all groups are shown in **(Fig 6A)**. The DEPs differed slightly between groups **(Fig 6B)**. Analysis of highly expressed proteins (HEPs) using the Jvenn database is shown in **(Fig 6C)**. There were 257, 43, 397, and 98 proteins identified only in the normal, HFF, Ov, and HFFOv groups, respectively. In this study we focused on the HFFOv group to look for proteins that may be associated with kidney disease. Gene ontology or functional categories based on the Uniprot database were used to analyze the proteins unique to the HFFOv group.

**Fig 7:**
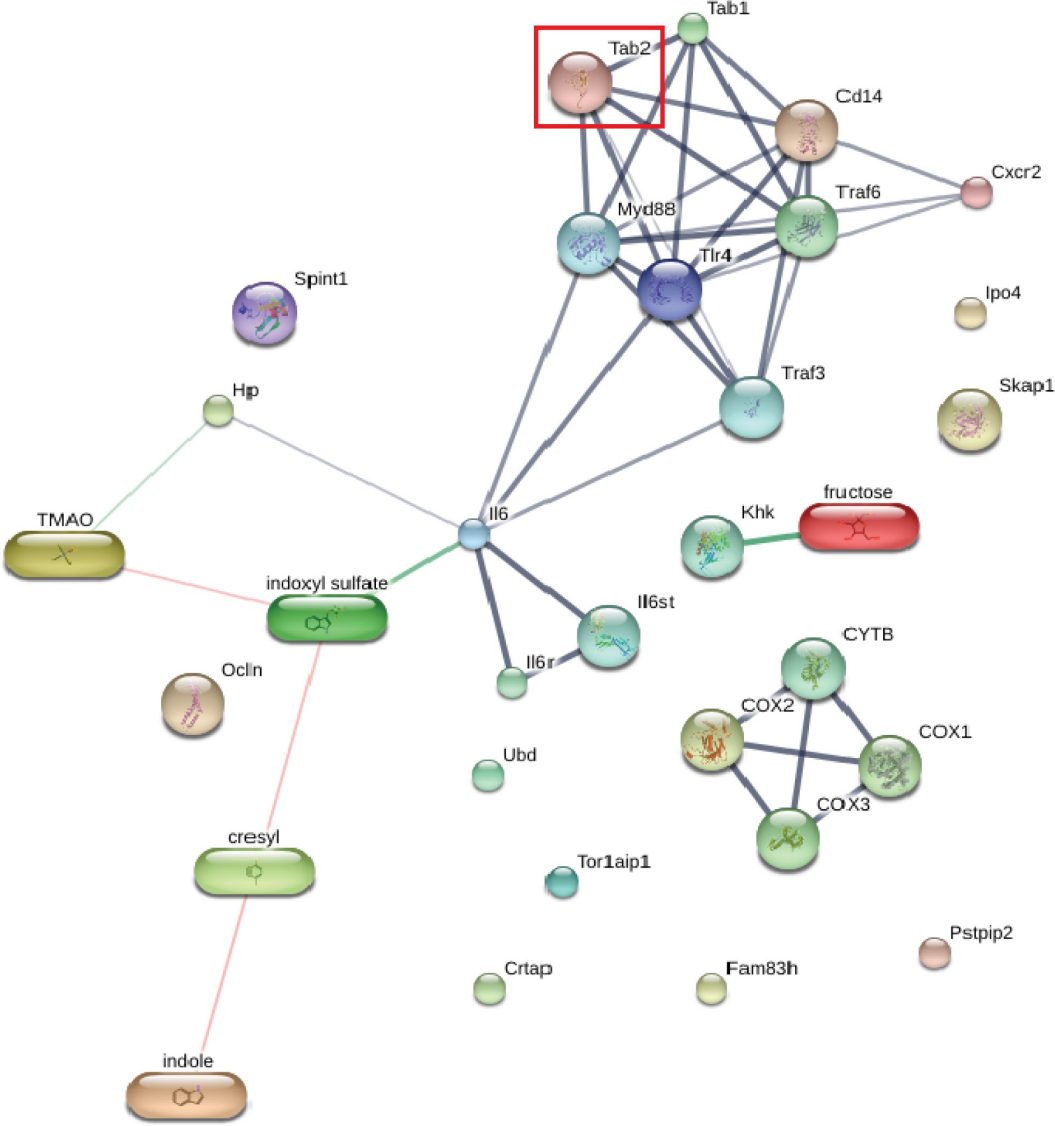
Protein-chemical interactions indicate inflammation and leaky gut. TGF-beta-activated kinase 1 and MAP3K7-binding protein 2 (Tab2) interacted directly with mitogen-activated protein kinase kinase kinase 7 interacting protein 1 (Tab1), TNF receptor-associated factor 6 (Traf6), Toll-like receptor 4 precursor (Tlr4), myeloid differentiation primary response protein MyD88 (Myd88), and monocyte differentiation antigen CD14 (Cd14). The thicker lines represent the highest confidence (0.900). The thin lines represent the medium confidence (0.400).

Gene ontology or functional category were identified for 46 HEPs: only 15 HEPs were associated with kidney structure and/or function, as shown in **S1 Table**. DEPs in the HFFOv group were selected for STITCH analysis. The results showed that TGF-beta-activated kinase 1 and MAP3K7-binding protein 2 (Tab2) interact with biomolecules involved in the inflammatory process, such as trimethylamine N-oxide (TMAO), indoxyl sulfate, p-cresol, indole, and IL-6. In addition, we found that Tab2 interacts with soluble CD14, which may promote a leaky gut (**Fig 7**).

## Discussion

Ingestion of high-fat, high-sugar (HFF) diets is associated with chronic kidney disease (CKD) [30]. We have previously reported that *O*. *viverrini* infection and HFF diet intake exacerbates liver pathology [31], which may in turn promote kidney disease [11]. However, the underlying mechanism is unclear. In this study we used the leftover specimens from the previous study. We employed the Illumina MiSeq sequencing platform, targeting the V3–V4 region of the 16S rRNA gene and analyzed the host proteome using LC-MS/MS. We first demonstrated that alterations in metagenomics and metaproteomics parameters are associated with kidney disease in hamsters given a combination of HFF diet and *O*. *viverrini* infection. The finding in an animal model might provide information for health awareness to limit kidney injury and intervention of kidney disease.

First, we investigated biochemical parameters of renal function, including BUN and SCr. Values of both these parameters were significantly lower in the Ov and HFFOv groups than in normal controls. The lower value of BUN may have been influenced by the 21% reduction of dietary protein in the groups receiving the HFF diet: BUN is a byproduct of protein metabolism [32]. Creatinine is synthesized from creatine, mainly in the liver. Creatine is synthesized from the transamination of the amino acids arginine, glycine, and methionine [33, 34]. Half of the lost creatinine can be replaced by a protein diet [35]. In our study, the nutrient ratio in the diet was adjusted by increasing the fat content. As a result, the protein content of the HFF diet was reduced. For this reason, serum levels BUN and creatinine levels could be lower in the HFF and HFFOv groups. The greater decrease in ACR observed in the HFFOv group compared to the other groups may be attributed to impaired liver function, as this group had more severe liver damage than the other groups [21]. In addition, the lower protein intake in the diet could possibly affect blood albumin levels [36]. Hence, we suggest that these biochemical parameters aren’t appropriate for use as biomarkers of kidney pathology in the HFF diet model.

We examined the histopathology of the kidneys by H&E staining and evaluated tissue fibrosis using picrosirius-red staining. The results showed that the combination of HFF diet and *O*. *viverrini* infection promoted renal injury, such as tubular dilatation, more than *O*. *viverrini* infection or HFF diet alone. This is consistent with previous studies showing that *O*. *viverrini* infection and/or HFF are associated with renal injury [10]. This result is supported by the significantly higher expression of KIM-1 and of fibrosis in the HFFOv group. Elevated levels of KIM-1 have been associated with inflammation and fibrosis in histopathological studies [37]. In addition, renal fibrosis is a reaction process similar to wound healing that occurs in renal injury. If the injury is severe, fibrosis may accumulate in the kidneys, leading to progression of renal dysfunction or eventually renal failure in the future [38].

Several recent studies have demonstrated a strong association between dysbiosis and CKD in both animal models and humans [39–42]. We have demonstrated the effects of changes in the gut in our animal model, showing that the combination of HFF diet and *O*. *viverrini* infection can alter the composition of gut microbiota in hamsters. The alpha diversity in the combination group (HFFOv) was higher than the in the HFF group. *Opisthorchis viverrini* infection might modulate the gut alteration [3, 4]. The results are supported by the increased microbial abundance in the HFFOv group when compared with the normal group, in which bacteria including *Ruminococcus*, *Lachnospiraceae*, *Desulfovibrionaceae*, and *Akkermansiaceae* were the most common. Similar to many previous reports, the microbiome in the gastrointestinal tract changed: *Lachnospiraceae*, *Ruminococcaceae*, and *Lactobacillaceae* increased, while *Porphyromonadaceae*, *Erysipelotrichaceae*, and *Eubacteriaceae* decreased in hamsters with fluke infestation [43]. Imbalances in the gut microbiota in CKD include changes in both abundance and composition, resulting in increased levels of *Lachnospiraceae*, *Enterobacteriaceae*, and *Ruminococcaceae* [44]. The family *Ruminococcaceae* (genus *Ruminococcus*) trended upward in the HFFOv group. Members of this family can produce pro-inflammatory factors that promote inflammatory bowel syndrome and promote CKD-associated molecules, such as glucorhamnan. This molecule induces dendritic cells to produce pro-inflammatory cytokines such as TNF-alpha [45, 46]. In addition, *Lachnospiraceae* and *Akkermansiaceae* also tended to increase in the HFFOv group. Similar to previous studies, *Akkermansiaceae* and *Lachnospiraceae* reduced in humans on low-protein diet and adenine-induced CKD rats, respectively [47, 48].

Our study determined the host fecal proteome associated with the HFF diet and *O*. *viverrini* infection. TGF-beta-activated kinase 1 and MAP3K7-binding protein 2 (Tab2) were upregulated. Overexpression of Tab2 is associated with the leaky-gut molecule (soluble CD14) [49] and also plays a key role in kidney disease via translocation of uremic toxins such as p-cresyl and indoxyl that promote CKD [50, 51]. Moreover, Tab2 protein is an important cellular signaling molecule that regulates inflammatory processes [52]. Increased inflammation in the gut due to alterations in the microbiome and proteome can lead to disruption of tight junctions in intestinal epithelial cells [53]. Chronic intestinal inflammation may further impair wound healing in the intestine and subsequently increase kidney fibrosis [54].

Recent research has shown that intestinal fibrosis can affect intestinal structure and function [55]. Consequently, loss of intestinal function due to inflammation and fibrosis may result in bacterial compounds and uremic toxins entering the bloodstream [56]. Gut-derived uremic toxins, including indoxyl sulfate, p-cresyl sulfate, and TMAO, can promote renal inflammation [57]. In addition, TMAO has been associated with increased tubulointerstitial fibrosis and progressive renal dysfunction [58]. Collectively, these findings suggest that the combination of HFF diet with *O*. *viverrini* infection is associated with renal disease through its effects on integrity of the intestinal barrier.

We acknowledge the limitation that our study was conducted in a hamster model. Databases for hamsters allowing identification of genes and proteins are still not sufficiently comprehensive. Further investigation in human patients is required. In addition, we used small sample sizes: larger sample sizes could increase the power of this finding. The expression of Tab2 protein should be further confirmed by the western-blot method.

## Conclusion

**Fig 8:**
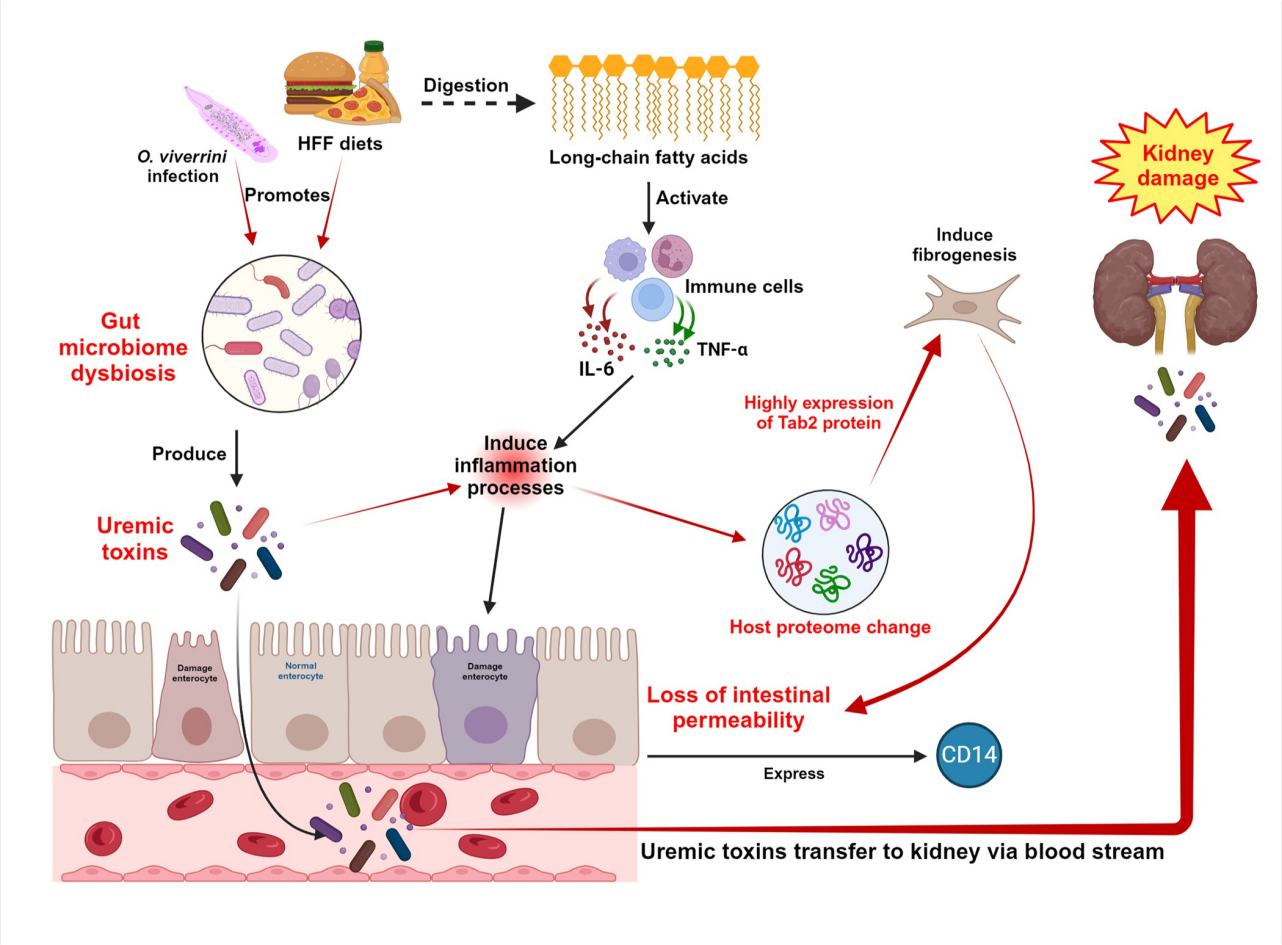
Schematic mechanism of the combination of HFF diet and *O*. *viverrini* infection promoting renal injury by alterations in the composition of the gut microbiota and host proteome. *Opisthorchis viverrini* infection causes gut dysbiosis and produces uremic toxin, inducing inflammation and a leaky gut. Ingestion of HFF diets activates immune cells and recruits inflammatory cells, that contribute to intestinal permeability via secretion of IL-6 and TNFα. Loss of intestinal permeability promote the expression of CD14. Expression of TGF-beta-activated kinase 1 and MAP3K7-binding protein 2 (Tab2) is increased during the inflammatory response, which contributes to the activation of fibrogenesis. In addition, uremic toxin leaks into the bloodstream, resulting in kidney damage. The image was reprinted from [https://www.biorender.com/] under a CC BY license number NM25VIFPY2, with permission from [BioRender], original copyright [2023] in **S2 File**.

The combination of HFF diet and chronic opisthorchiasis may promote kidney injury via the alteration of gut microbiome and host proteome in hamsters as shown in **Fig 8**. Metagenomics and metaproteomics revealed the high expression of Tab2 protein and leaky gut molecules such as soluble CD14, suggesting that HFF diet and *O*. *viverrini* infection might induce gut-derived uremic toxins and leaky gut, eventually leading to kidney injury/or pathology. We suggest that chronic opisthorchiasis and consumption of HFF diet promotes kidney disease in this way. The finding of this study may suggest a strategy for earlier treatment and mitigation of CKD progression beyond the early stages.

## Supporting information

S1 Table Gene ontology proteins associated with kidney disease in feces of hamster in HFFOv group. (PDF)

S1 Fig H&E staining for the EGTI histology scoring system (tubular, endothelial, glomerular, and tubulointerstitial) for the severity of kidney disease. (TIFF)

S2 Fig Immunohistochemical staining for kidney-injury molecule-1 (KIM-1) expression in the negative and positive controls. (TIF)

S3 Fig Immunohistochemical staining for kidney-injury molecule-1 (KIM-1) expression in the experimental groups. (TIF)

S1 File Microbiome diversity analysis pipeline. (PDF)

S2 File CC BY license number from Biorender. (PDF)

## References

[1] Kovesdy CP. Epidemiology of chronic kidney disease: an update 2022. Kidney International Supplements. 2022;12(1):7–11. doi: 10.1016/j.kisu.2021.11.003 35529086 PMC9073222

[2] Cha’on U, Tippayawat P, Sae-Ung N, Pinlaor P, Sirithanaphol W, Theeranut A, et al. High prevalence of chronic kidney disease and its related risk factors in rural areas of Northeast Thailand. Scientific Reports. 2022;12(1):18188. doi: 10.1038/s41598-022-22538-w 36307490 PMC9616930

[3] Itthitaetrakool U, Pinlaor P, Pinlaor S, Chomvarin C, Dangtakot R, Chaidee A, et al. Chronic *Opisthorchis viverrini* infection changes the liver microbiome and promotes *Helicobacter* growth. PLoS One. 2016;11(11):e0165798. 27806126 10.1371/journal.pone.0165798 PMC5091914

[4] Pakharukova MY, Lishai EA, Zaparina O, Baginskaya NV, Hong SJ, Sripa B, et al. *Opisthorchis viverrini*, *Clonorchis sinensis* and *Opisthorchis felineus* liver flukes affect mammalian host microbiome in a species-specific manner. PLoS Negl Trop Dis. 2023;17(2):e0011111. 36780567 10.1371/journal.pntd.0011111 PMC9956601

[5] Pongking T, Haonon O, Dangtakot R, Onsurathum S, Jusakul A, Intuyod K, et al. A combination of monosodium glutamate and high-fat and high-fructose diets increases the risk of kidney injury, gut dysbiosis and host-microbial co-metabolism. Plos one. 2020;15(4):e0231237. doi: 10.1371/journal.pone.0231237 32267892 PMC7141667

[6] Wehedy E, Shatat IF, Al Khodor S. The human microbiome in chronic kidney disease: a double-edged sword. Front Med (Lausanne). 2021;8:790783. doi: 10.3389/fmed.2021.790783 35111779 PMC8801809

[7] de Castro UGM, dos Santos RASAS, Silva ME, e Lima WG, Campagnole-Santos MJ, Alzamora AC. Age-dependent effect of high-fructose and high-fat diets on lipid metabolism and lipid accumulation in liver and kidney of rats. Lipids in Health and Disease. 2013;12(1):136. doi: 10.1186/1476-511X-12-136 24044579 PMC3849586

[8] Dissard R, Klein J, Caubet C, Breuil B, Siwy J, Hoffman J, et al. Long term metabolic syndrome induced by a high fat high fructose diet leads to minimal renal injury in C57BL/6 mice. PLoS One. 2013;8(10):e76703. doi: 10.1371/journal.pone.0076703 24098551 PMC3789664

[9] Boonpucknavig S, Boonpucknavig V, Tanvanich S, Doungchawee G, Thamavit W. Development of immune-complex glomerulonephritis and amyloidosis in Syrian golden hamsters infected with *Opisthorchis viverrini*. J Med Assoc Thai. 1992;75 Suppl 1:7–19. 1402485

[10] Haonon O, Liu Z, Dangtakot R, Intuyod K, Pinlaor P, Puapairoj A, et al. *Opisthorchis viverrini* infection induces metabolic and fecal microbial disturbances in association with liver and kidney pathologies in hamsters. J Proteome Res. 2021;20(8):3940–51. 34270897 10.1021/acs.jproteome.1c00246

[11] Haonon O, Liu Z, Dangtakot R, Pinlaor P, Puapairoj A, Cha’on U, et al. *Opisthorchis viverrini* infection induces metabolic disturbances in hamsters fed with high fat/high fructose diets: implications for liver and kidney pathologies. J. Nutr. Biochem. 2022;107:109053. 35643287 10.1016/j.jnutbio.2022.109053

[12] Andrade-Oliveira V, Foresto-Neto O, Watanabe IKM, Zatz R, Câmara NOS. Inflammation in renal diseases: new and old players. Front Pharmacol. 2019;10:1192. doi: 10.3389/fphar.2019.01192 31649546 PMC6792167

[13] Mihai S, Codrici E, Popescu ID, Enciu AM, Albulescu L, Necula LG, et al. Inflammation-related mechanisms in chronic kidney disease prediction, progression, and outcome. J Immunol Res. 2018;2018:2180373. doi: 10.1155/2018/2180373 30271792 PMC6146775

[14] Velmurugan G, Dinakaran V, Rajendhran J, Swaminathan K. Blood microbiota and circulating microbial metabolites in diabetes and cardiovascular disease. Trends Endocrinol Metab. 2020;31(11):835–47. doi: 10.1016/j.tem.2020.01.013 33086076

[15] Hsu CN, Yang HW, Hou CY, Chang-Chien GP, Lin S, Tain YL. Maternal adenine-induced chronic kidney disease programs hypertension in adult male rat offspring: implications of nitric oxide and gut microbiome derived metabolites. Int J Mol Sci. 2020;21(19). doi: 10.3390/ijms21197237 33008046 PMC7583952

[16] Lichtman JS, Marcobal A, Sonnenburg JL, Elias JE. Host-centric proteomics of stool: a novel strategy focused on intestinal responses to the gut microbiota. Mol Cell Proteomics. 2013;12(11):3310–8. doi: 10.1074/mcp.M113.029967 23982161 PMC3820941

[17] Starr AE, Deeke SA, Li L, Zhang X, Daoud R, Ryan J, et al. Proteomic and metaproteomic approaches to understand host-microbe interactions. Anal Chem. 2018;90(1):86–109. doi: 10.1021/acs.analchem.7b04340 29061041

[18] Moresco RN, De Carvalho JAM. Applying proteomics to diagnosis of diabetic kidney disease. Expert Rev Proteomics. 2017;14(10):841–3. doi: 10.1080/14789450.2017.1378100 28893107

[19] Dubin RF, Rhee EP. Proteomics and metabolomics in kidney disease, including Insights into etiology, treatment, and prevention. Clin J Am Soc Nephrol. 2020;15(3):404–11. doi: 10.2215/CJN.07420619 31636087 PMC7057308

[20] Zacchia M, Marchese E, Trani EM, Caterino M, Capolongo G, Perna A, et al. Proteomics and metabolomics studies exploring the pathophysiology of renal dysfunction in autosomal dominant polycystic kidney disease and other ciliopathies. Nephrol Dial Transplant. 2020;35(11):1853–61. doi: 10.1093/ndt/gfz121 31219585

[21] Sitthirach C, Charoensuk L, Pairojkul C, Chaidee A, Intuyod K, Pongking T, et al. Curcumin-loaded nanocomplexes ameliorate the severity of nonalcoholic steatohepatitis in hamsters infected with *Opisthorchis viverrini*. Plos one. 2022;17(9):e0275273. doi: 10.1371/journal.pone.0275273 36166461 PMC9514634

[22] Khalid U, Pino-Chavez G, Nesargikar P, Jenkins RH, Bowen T, Fraser DJ, et al. Kidney ischaemia reperfusion injury in the rat: the EGTI scoring system as a valid and reliable tool for histological assessment. Histol. Histopathol. 2016;3(1):1.

[23] Yacoub R, Habib H, Lahdo A, Al Ali R, Varjabedian L, Atalla G, et al. Association between smoking and chronic kidney disease: a case control study. BMC public health. 2010;10(1):1–6. doi: 10.1186/1471-2458-10-731 21108832 PMC3004836

[24] Nemmar A, Karaca T, Beegam S, Yuvaraju P, Yasin J, Ali BH. Lung oxidative stress, DNA damage, apoptosis, and fibrosis in adenine-induced chronic kidney disease in mice. Front Physiol. 2017;8:896. doi: 10.3389/fphys.2017.00896 29218013 PMC5703828

[25] Rangan GK, Tesch GH. Quantification of renal pathology by image analysis. Nephrology (Carlton). 2007;12(6):553–8. doi: 10.1111/j.1440-1797.2007.00855.x 17995580

[26] Özkurt E, Fritscher J, Soranzo N, Ng DYK, Davey RP, Bahram M, et al. LotuS2: an ultrafast and highly accurate tool for amplicon sequencing analysis. Microbiome. 2022;10(1):176. doi: 10.1186/s40168-022-01365-1 36258257 PMC9580208

[27] Hildebrand F, Tadeo R, Voigt AY, Bork P, Raes J. LotuS: an efficient and user-friendly OTU processing pipeline. Microbiome. 2014;2(1):30. doi: 10.1186/2049-2618-2-30 27367037 PMC4179863

[28] White JR, Nagarajan N, Pop M. Statistical methods for detecting differentially abundant features in clinical metagenomic samples. PLoS Comput Biol. 2009;5(4):e1000352. doi: 10.1371/journal.pcbi.1000352 19360128 PMC2661018

[29] Lowry OH, Rosebrough NJ, Farr AL, Randall RJ. Protein measurement with the Folin phenol reagent. J Biol Chem. 1951;193(1):265–75. 14907713

[30] Asghari G, Momenan M, Yuzbashian E, Mirmiran P, Azizi F. Dietary pattern and incidence of chronic kidney disease among adults: a population-based study. Nutr Metab (Lond). 2018;15:88. doi: 10.1186/s12986-018-0322-7 30564279 PMC6296119

[31] Chaidee A, Onsurathum S, Intuyod K, Haonon O, Pannangpetch P, Pongchaiyakul C, et al. *Opisthorchis viverrini* infection augments the severity of nonalcoholic fatty liver disease in high-fat/high-fructose diet-fed hamsters. Am J Trop Med Hyg. 2019;101(5):1161–9. 31482785 10.4269/ajtmh.19-0442 PMC6838561

[32] Xue Y, Daniels LB, Maisel AS, Iqbal N. Cardiac biomarkers. Reference Module in Biomedical Sciences: Elsevier; 2014.

[33] Salazar JH. Overview of urea and creatinine. Laboratory medicine. 2014;45(1):e19–e20.

[34] Thongprayoon C, Cheungpasitporn W, Kashani K. Serum creatinine level, a surrogate of muscle mass, predicts mortality in critically ill patients. J Thorac Dis. 2016;8(5):E305–11. doi: 10.21037/jtd.2016.03.62 27162688 PMC4842835

[35] Brosnan JT, da Silva RP, Brosnan ME. The metabolic burden of creatine synthesis. Amino Acids. 2011;40(5):1325–31. doi: 10.1007/s00726-011-0853-y 21387089

[36] Jeejeebhoy KN. Nutritional assessment. In: Johnson LR, editor. Encyclopedia of Gastroenterology. New York: Elsevier; 2004. p. 759–66.

[37] van Timmeren MM, van den Heuvel MC, Bailly V, Bakker SJ, van Goor H, Stegeman CA. Tubular kidney injury molecule-1 (KIM-1) in human renal disease. J Pathol. 2007;212(2):209–17. doi: 10.1002/path.2175 17471468

[38] Lee J, Kim K, Kim S. Chapter 5—kidney on chips. In: Doh J, Fletcher D, Piel M, editors. Methods in Cell Biology. 146: Academic Press; 2018. p. 85–104. 10.1016/bs.mcb.2018.06.001 30037468

[39] Huang L, Liu B, Liu Z, Feng W, Liu M, Wang Y, et al. Gut microbiota exceeds cervical microbiota for early diagnosis of endometriosis. Front Cell Infect Microbiol. 2021;11:788836. doi: 10.3389/fcimb.2021.788836 34950610 PMC8688745

[40] Wang X, Yang S, Li S, Zhao L, Hao Y, Qin J, et al. Aberrant gut microbiota alters host metabolome and impacts renal failure in humans and rodents. Gut. 2020;69(12):2131–42. doi: 10.1136/gutjnl-2019-319766 32241904 PMC7677483

[41] Ramezani A, Raj DS. The gut microbiome, kidney disease, and targeted interventions. J Am Soc Nephrol. 2014;25(4):657–70. doi: 10.1681/ASN.2013080905 24231662 PMC3968507

[42] Noce A, Marchetti M, Marrone G, Di Renzo L, Di Lauro M, Di Daniele F, et al. Link between gut microbiota dysbiosis and chronic kidney disease. Eur Rev Med Pharmacol Sci. 2022;26(6):2057–74. doi: 10.26355/eurrev_202203_28354 35363356

[43] Plieskatt JL, Deenonpoe R, Mulvenna JP, Krause L, Sripa B, Bethony JM, et al. Infection with the carcinogenic liver fluke *Opisthorchis viverrini* modifies intestinal and biliary microbiome. Faseb j. 2013;27(11):4572–84. 23925654 10.1096/fj.13-232751 PMC3804743

[44] Sampaio-Maia B, Simões-Silva L, Pestana M, Araujo R, Soares-Silva IJ. The role of the gut microbiome on chronic kidney disease. Adv Appl Microbiol. 2016;96:65–94. doi: 10.1016/bs.aambs.2016.06.002 27565581

[45] Bhargava S, Merckelbach E, Noels H, Vohra A, Jankowski J. Homeostasis in the gut microbiota in chronic kidney disease. Toxins (Basel). 2022;14(10). doi: 10.3390/toxins14100648 36287917 PMC9610479

[46] Henke MT, Kenny DJ, Cassilly CD, Vlamakis H, Xavier RJ, Clardy J. *Ruminococcus gnavus*, a member of the human gut microbiome associated with Crohn’s disease, produces an inflammatory polysaccharide. Proceedings of the National Academy of Sciences. 2019;116(26):12672–7. 10.1073/pnas.1904099116 PMC6601261 31182571

[47] Lai S, Molfino A, Testorio M, Perrotta AM, Currado A, Pintus G, et al. Effect of low-protein diet and inulin on microbiota and clinical parameters in patients with chronic kidney disease. Nutrients. 2019;11(12). doi: 10.3390/nu11123006 31818021 PMC6950025

[48] Zhang ZM, Yang L, Wan Y, Liu C, Jiang S, Shang EX, et al. Integrated gut microbiota and fecal metabolomics reveal the renoprotective effect of Rehmanniae Radix Preparata and Corni Fructus on adenine-induced CKD rats. J Chromatogr B Analyt Technol Biomed Life Sci. 2021;1174:122728. doi: 10.1016/j.jchromb.2021.122728 33975272

[49] Al-Ayadhi L, Zayed N, Bhat RS, Moubayed NMS, Al-Muammar MN, El-Ansary A. The use of biomarkers associated with leaky gut as a diagnostic tool for early intervention in autism spectrum disorder: a systematic review. Gut Pathog. 2021;13(1):54. doi: 10.1186/s13099-021-00448-y 34517895 PMC8439029

[50] Lau WL, Kalantar-Zadeh K, Vaziri ND. The gut as a source of inflammation in chronic kidney disease. Nephron. 2015;130(2):92–8. doi: 10.1159/000381990 25967288 PMC4485546

[51] Anders HJ, Andersen K, Stecher B. The intestinal microbiota, a leaky gut, and abnormal immunity in kidney disease. Kidney Int. 2013;83(6):1010–6. doi: 10.1038/ki.2012.440 23325079

[52] Bettermann K, Vucur M, Haybaeck J, Koppe C, Janssen J, Heymann F, et al. TAK1 suppresses a NEMO-dependent but NF-kappaB-independent pathway to liver cancer. Cancer Cell. 2010;17(5):481–96. doi: 10.1016/j.ccr.2010.03.021 20478530

[53] Lechuga S, Ivanov AI. Disruption of the epithelial barrier during intestinal inflammation: quest for new molecules and mechanisms. Biochim Biophys Acta Mol Cell Res. 2017;1864(7):1183–94. doi: 10.1016/j.bbamcr.2017.03.007 28322932 PMC5507344

[54] Zhan S, Li N, Liu C, Mao R, Wu D, Li T, et al. Intestinal fibrosis and gut microbiota: clues from other organs. Front Microbiol. 2021;12:694967. doi: 10.3389/fmicb.2021.694967 34335525 PMC8322786

[55] Wang Y, Huang B, Jin T, Ocansey DKW, Jiang J, Mao F. Intestinal fibrosis in inflammatory bowel disease and the prospects of mesenchymal stem eell therapy. Front Immunol. 2022;13:835005. 35370998 10.3389/fimmu.2022.835005 PMC8971815

[56] Amini Khiabani S, Asgharzadeh M, Samadi Kafil H. Chronic kidney disease and gut microbiota. Heliyon. 2023;9(8):e18991. doi: 10.1016/j.heliyon.2023.e18991 37609403 PMC10440536

[57] Lau Wei L, Savoj J, Nakata Michael B, Vaziri Nosratola D. Altered microbiome in chronic kidney disease: systemic effects of gut-derived uremic toxins. Clinical Science. 2018;132(5):509–22. doi: 10.1042/CS20171107 29523750

[58] Zeng Y, Guo M, Fang X, Teng F, Tan X, Li X, et al. Gut microbiota-derived trimethylamine N-oxide and kidney function: a systematic review and meta-analysis. Advances in Nutrition. 2021;12(4):1286–304. doi: 10.1093/advances/nmab010 33751019 PMC8321840

