# Supplementary material for: Metagenomics and metaproteomics alterations are associated with kidney disease in opisthorchiasis hamsters fed a high-fat and high-fructose diet
Source: PLoS One. 2024 May 30;19(5):e0301907. doi: 10.1371/journal.pone.0301907 (PMC11139331; doi:10.1371/journal.pone.0301907)
Supplement: S1 Table — (PDF) [file pone.0301907.s001.pdf]

S1 Table. Gene ontology proteins associated with kidney disease in feces of hamster in HFFOv group.

Protein IDs	Protein name	Gene Names	Gene Ontology (biological process)	Gene Ontology (molecular function)
A0A1U8CC24	TGF-beta-activated kinase 1 and MAP3K7-binding protein 3 isoform X1	Tab3	phosphorylation [GO:0016310]	kinase activity [GO:0016301]; metal ion binding [GO:0046872]
A0A1U7Q9R2	Hedgehog protein	Dhh	cell-cell signaling [GO:0007267]; intein-mediated protein splicing [GO:0016539]; Leydig cell differentiation [GO:0033327]; male sex determination [GO:0030238]; osteoblast differentiation [GO:0001649]; positive regulation of smoothened signaling pathway [GO:0045880]; protein auto processing [GO:0016540]; regulation of steroid biosynthetic process [GO:0050810]; smoothened signaling pathway [GO:0007224]; spermatid development [GO:0007286]	calcium ion binding [GO:0005509]; patched binding [GO:0005113]; peptidase activity [GO:0008233]; transferase activity [GO:0016740]; zinc ion binding [GO:0008270]
A0A3Q0D3N6	Protein unc-13 homolog B isoform X6	Unc13b	modulation of chemical synaptic transmission [GO:0050804]; synaptic vesicle exocytosis [GO:0016079]	calcium ion binding [GO:0005509]; diacylglycerol binding [GO:0019992]; phospholipid binding [GO:0005543]

A0A1U7RHX1	Proto-oncogene serine/threonine-protein kinase mos	Mos	protein phosphorylation [GO:0006468]	ATP binding [GO:0005524]; protein serine/threonine kinase activity [GO:0004674]
A0A3Q0CMN0	MAPK/MAK/MRK Overlapping kinase isoform X3	Mok	protein phosphorylation [GO:0006468]	ATP binding [GO:0005524]; protein serine/threonine kinase activity [GO:0004674]
A0A1U7QC34	Nucleoside diphosphate kinase (EC 2.7.4.6)	LOC101838863	CTP biosynthetic process [GO:0006241]; GTP biosynthetic process [GO:0006183]; phosphorylation [GO:0016310]; UTP biosynthetic process [GO:0006228]	ATP binding [GO:0005524]; nucleoside diphosphate kinase activity [GO:0004550]
A0A3Q0CW60	Probable glutamate--tRNA ligase, mitochondrial (EC 6.1.1.17) (Glutamyl-tRNA synthetase)	Ears2	glutamyl-tRNA aminoacylation [GO:0006424]	ATP binding [GO:0005524]; glutamate-tRNA ligase activity [GO:0004818]; tRNA binding [GO:0000049]; zinc ion binding [GO:0008270]
A0A3Q0CRW4	Diacylglycerol kinase (DAG kinase) (EC 2.7.1.107)	Dgkh	glycerolipid metabolic process [GO:0046486]; protein kinase C-activating G protein-coupled receptor signaling pathway [GO:0007205]	ATP binding [GO:0005524]; diacylglycerol kinase activity [GO:0004143];

				metal ion binding [GO:0046872]
A0A1U7R335	Tyrosine-protein kinase receptor (EC 2.7.10.1)	Ntrk2	cellular response to amino acid stimulus [GO:0071230]; central nervous system neuron development [GO:0021954]; glutamate secretion [GO:0014047]; learning [GO:0007612]; negative regulation of amyloid-beta formation [GO:1902430]; negative regulation of anoikis [GO:2000811]; neuron migration [GO:0001764]; neuronal action potential propagation [GO:0019227]; oligodendrocyte differentiation [GO:0048709]; positive regulation of gene expression [GO:0010628]; positive regulation of neuron projection development [GO:0010976]; positive regulation of peptidyl-serine phosphorylation [GO:0033138]; positive regulation of synapse assembly [GO:0051965]; regulation of GTPase activity [GO:0043087]; regulation of protein kinase B signaling [GO:0051896]; retinal rod cell development [GO:0046548]; trans-synaptic signaling by BDNF, modulating synaptic transmission [GO:0099183]; trans-synaptic signaling by neuropeptide, modulating	ATP binding [GO:0005524]; brain-derived neurotrophic factor binding [GO:0048403]; brain-derived neurotrophic factor receptor activity [GO:0060175]; protease binding [GO:0002020]

			synaptic transmission [GO:0099551]; vasculogenesis [GO:0001570]	
A0A3Q0CT59	E3 ubiquitin-protein ligase CBL (EC 2.3.2.27)	Cblb	cell surface receptor signaling pathway [GO:0007166]; protein ubiquitination [GO:0016567]; regulation of signaling [GO:0023051]	aspartyltransferase activity [GO:0047690]; calcium ion binding [GO:0005509]; phosphotyrosine residue binding [GO:0001784]; ubiquitin-protein transferase activity [GO:0004842]
A0A1U7Q4S6	Alpha-2C adrenergic receptor (Alpha-2C adrenoreceptor)	Adra2c	activation of protein kinase B activity [GO:0032148]; platelet activation [GO:0030168]; positive regulation of MAPK cascade [GO:0043410]; positive regulation of neuron differentiation [GO:0045666]; receptor transactivation [GO:0035624]; regulation of smooth muscle contraction [GO:0006940]; regulation of vasoconstriction [GO:0019229]	alpha-2A adrenergic receptor binding [GO:0031694]; alpha2-adrenergic receptor activity [GO:0004938]; epinephrine binding [GO:0051379]; protein heterodimerization activity [GO:0046982]; protein homodimerization activity [GO:0042803]
A0A1U7Q3V8	Protein phosphatase 1	Ppp1r42	regulation of phosphatase activity [GO:0010921]	actin binding [GO:0003779]; dynein complex binding

	regulatory subunit 42 isoform X1			[GO:0070840]; tubulin binding [GO:0015631]
A0A1U8CLP9	Phosphodiesterase (EC 3.1.4.-)	Pde1a	signal transduction [GO:0007165]	3',5'-cyclic-AMP phosphodiesterase activity [GO:0004115]; 3',5'-cyclic-GMP phosphodiesterase activity [GO:0047555]; metal ion binding [GO:0046872]
A0A1U7Q4L4	Glycerol-3-phosphate acyltransferase 2, mitochondrial	Gpat2	glycerol-3-phosphate metabolic process [GO:0006072]; phosphatidic acid biosynthetic process [GO:0006654]; piRNA processing [GO:0034587]; triglyceride biosynthetic process [GO:0019432]	1-acylglycerol-3-phosphate O-acyltransferase activity [GO:0003841]; glycerol-3-phosphate O-acyltransferase activity [GO:0004366]
A0A1U8C335	[heparan sulfate]-glucosamine N-sulfotransferase (EC 2.8.2.8)	LOC101840021	heparan sulfate proteoglycan biosynthetic process, polysaccharide chain biosynthetic process [GO:0015014]; heparin biosynthetic process [GO:0030210]	[heparan sulfate]-glucosamine N-sulfotransferase activity [GO:0015016]; heparan sulfate N-deacetylase activity [GO:0102140]
