# Supplementary figures and images for: Metagenomics and metaproteomics alterations are associated with kidney disease in opisthorchiasis hamsters fed a high-fat and high-fructose diet
Source: PLoS One. 2024 May 30;19(5):e0301907. doi: 10.1371/journal.pone.0301907 (PMC11139331; doi:10.1371/journal.pone.0301907)

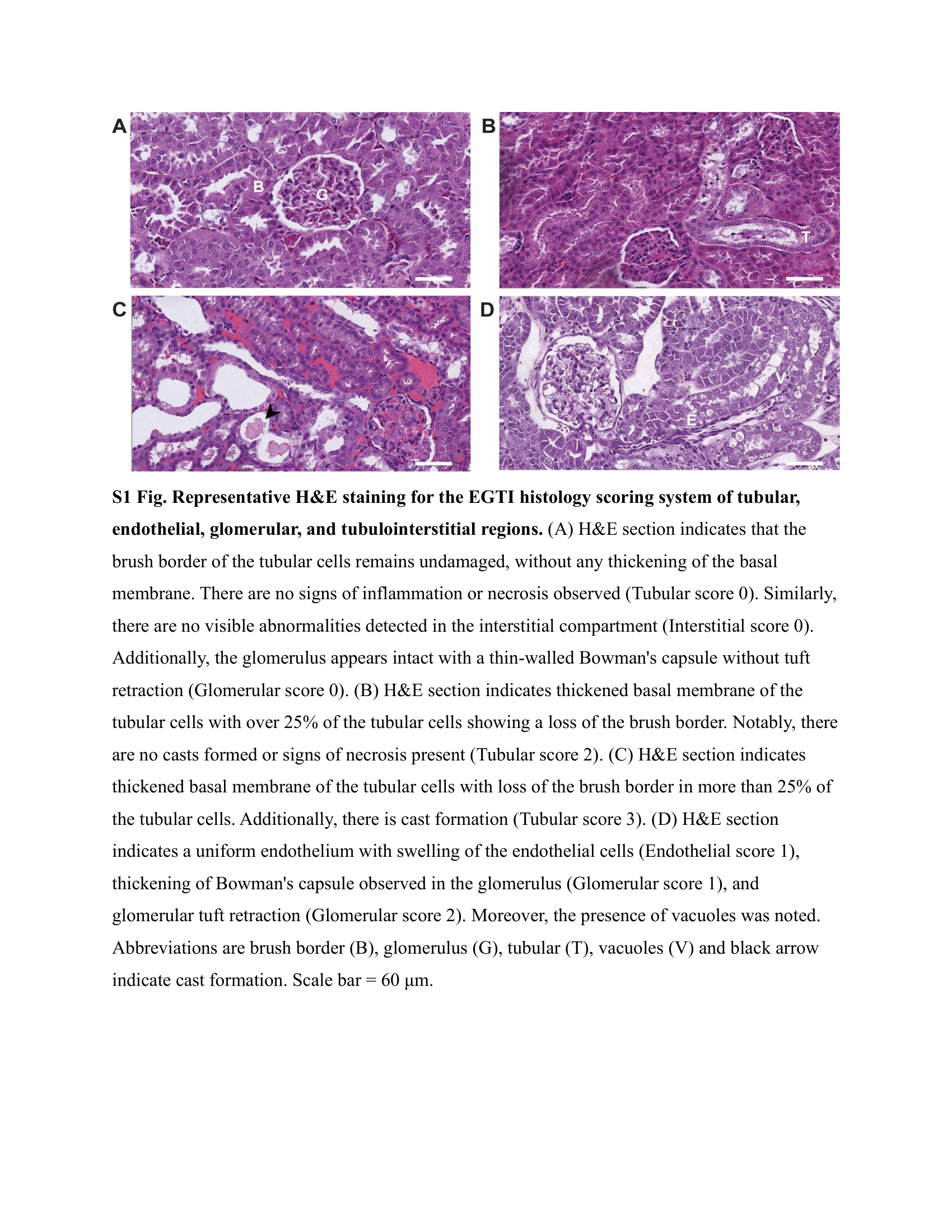

Supplement: S1 Fig — (TIFF) [file pone.0301907.s002.tiff]

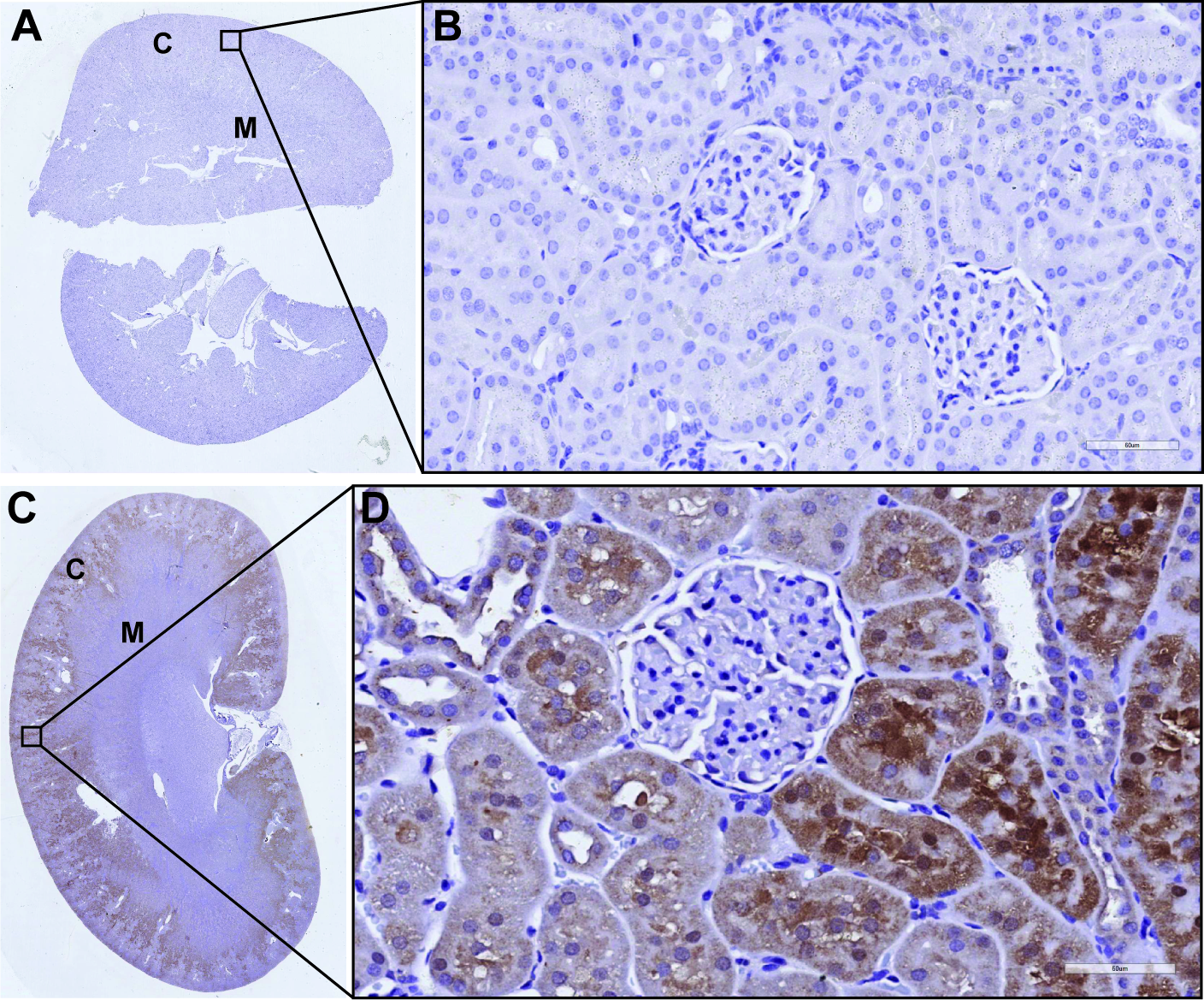

Supplement: S2 Fig — (TIF) [file pone.0301907.s003.tif]

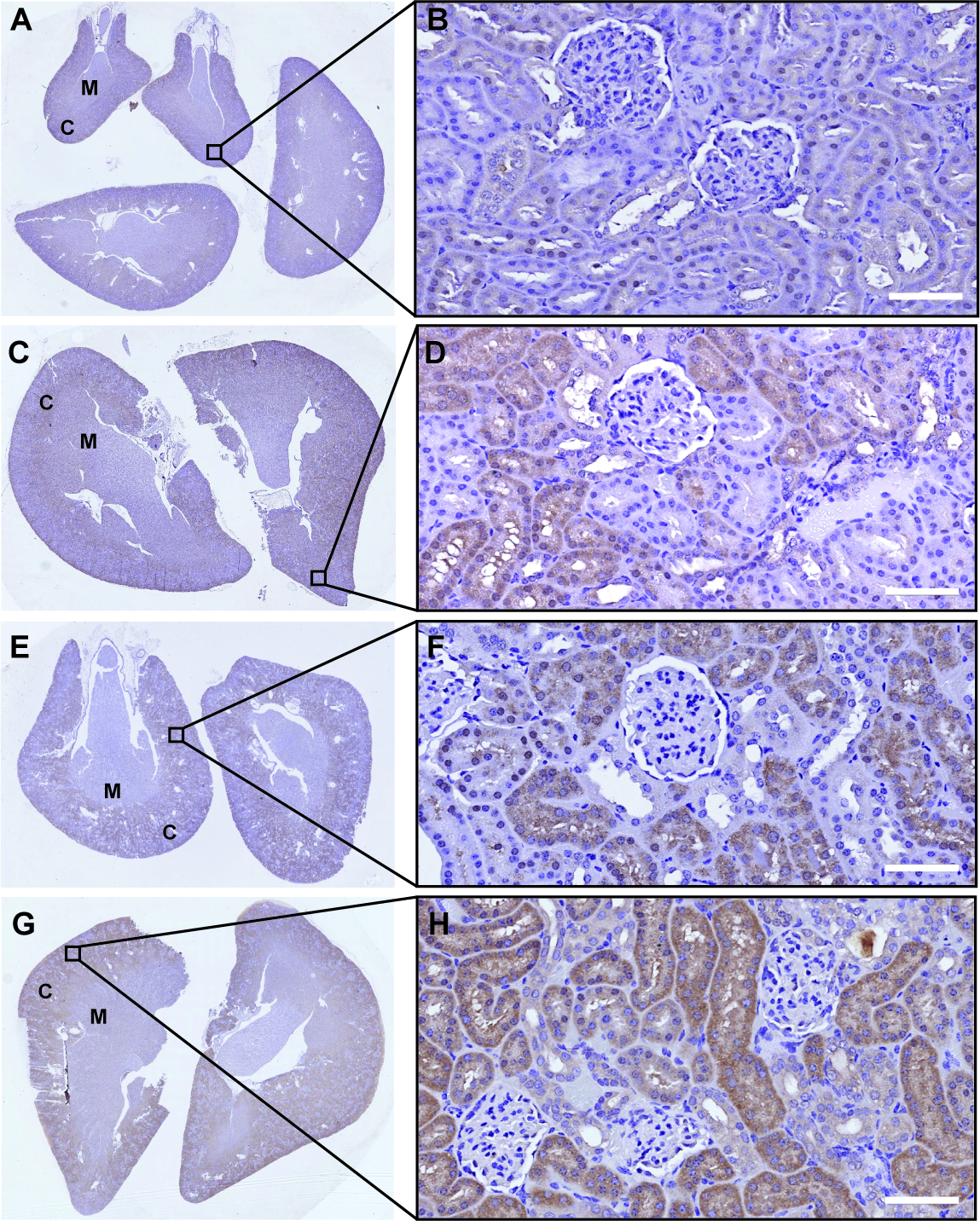

Supplement: S3 Fig — (TIF) [file pone.0301907.s004.tif]
