# Supplementary material for: Metagenomics and metaproteomics alterations are associated with kidney disease in opisthorchiasis hamsters fed a high-fat and high-fructose diet
Source: PLoS One. 2024 May 30;19(5):e0301907. doi: 10.1371/journal.pone.0301907 (PMC11139331; doi:10.1371/journal.pone.0301907)
Supplement: S1 File — (PDF) [file pone.0301907.s005.pdf]

Microbiome diversity analysis was calculated using R software version 4.3.0. We used all the libraries in this study including library(tidyverse), library(phyloseq), library(DESeq2), library(dplyr), library(vegan), library(stats), library(multcomp), library(ggpubr), library(rstatix) and library(metagMisc).

The bioinformatic pipeline used of microbiome analysis was performed as following steps.

1. Alpha diversity, indicating species diversity within a sample, was evaluated by Shannon and Simpson indices, using phyloseq package version 1.44.0; ggboxplot were used for alpha diversity plotting and statistical comparison by ggpubur package version 0.6.0.

2. Beta diversity analysis, which examines differences in species composition among samples, The PCoA of Bray-Curtis distance was computed using the vegan package version 2.6.4, along with weighted UniFrac values calculated using the UniFrac method from the phyloseq package version 1.44.0. Statistical comparison between sample groups was performed using Permutational multivariate analysis of variance (PERMANOVA). Principal component analysis (PCA) plots were generated using cmdscale from the stats package version 4.3.0.

3. Relative abundance was calculated using the phyloseq package version 1.44.0, and stacked bar-plots were created based on relative abundance values, with prevalence filtering: 10%, using ggplot2 version 3.4.4.

4. Heat map was constructed based on the relative abundance value of microbiota which calculated to row Z score, was generated using the pheatmap package version 1.0.12.
